# EHMTI-0021. The possible effect of telmisartan on the blood-brain barrier transport of verapamil

**DOI:** 10.1186/1129-2377-15-S1-F28

**Published:** 2014-09-18

**Authors:** PL Saaby, P Tfelt-Hansen

**Affiliations:** 1 Faculty of Health and Medical Sciences University of Copenhagen, Bioneer:FARMA, Copenhagen, Denmark; 2 Glostrup Hospital University of Copenhagen, Danish Headache Center, Glostrup, Denmark

## Introduction

The high doses of verapamil (480 to 960 mg per day) needed in cluster headache prevention are most likely due to a need for the drug to exert an effect in the CNS. Verapamil is cell permeable due to its lipophilic nature, but is also, at the same time, a substrate for the BBB efflux pump P-glycoprotein (P-gp). If the efflux of verapamil could be selectively inhibited the blood concentration of verapamil needed for an CNS effect could probably be decreased.

In an in vitro study using a P388/dx cell line, “telmisartan was identified as one of the most potent inhibitors of P-gp currently known” [Biopharm. Drug Disp. 2010; 31: 150-161.]. We therefore investigated whether telmisartan could modulate the transport of verapamil in an in vitro model of the BBB.

## Methods and results

In bi-directional transport experiments (n=6), telmisartan (2.4 µM) increased both uptake and permeability of verapamil in MDCK-II MDR1 cells cultured on permeable supports. Telmisartan also reduced the ratio between brain to blood and blood to brain transport; indicating a telmisartan-mediated modulation of verapamil efflux. Telmisartan did not affect efflux transport of digoxin.

## Discussion

The main result of this in vitro of the transport across the BBB was the indication of a telmisartan-mediated inhibition of verapamil efflux transport. The lack of effect of telmisartan on efflux transport of digoxin might indicate that the observed effects on verapamil efflux are not related to inhibition of P-gp or that telmisartan does not block the digoxin-binding site at P-gp.

No conflict of interest.

